# Supplementary material for: Modelling the effects of emotional engagement and peer interaction on the continuous intention to use asynchronous e-learning
Source: PeerJ Comput Sci. 2024 Apr 22;10:e1990. doi: 10.7717/peerj-cs.1990 (PMC11057566; doi:10.7717/peerj-cs.1990)
Supplement: Supplemental Information 2 [file peerj-cs-10-1990-s002.docx]

**Questionnaires**

Perceived usefulness (adapted from Davis (1989))

PU1 Learning using asynchronous e-learning will improve my work.

PU2 Learning using asynchronous e-learning will enhance my effectiveness.

PU3 Learning using asynchronous e-learning will increase my productivity.

Perceived ease of use (adapted from Davis (1989))

PEU1 My interaction with asynchronous e-learning is clear and understandable.

PEU2 I find it easy to learn through asynchronous e-learning and to do what I want it to do.

PEU3 I find asynchronous e-learning easy to use.

Behavioural intention (adapted from Davis (1989))

BI1 I will use asynchronous e-learning in future.

BI2 I plan to use the asynchronous e-learning often

BI3 Assuming that I have access to an asynchronous e-learning, I intend to use it

The emotional engagement (Wara et al, (2018).

I feel relaxed with learning In asynchronous e-learning.

If I could choose a learning style, I would choose asynchronous e-learning.

I am never bored in asynchronous e-learning.

I have never considered dropping out from asynchronous e-learning.

I can participate and suggest convenient materials for learning in asynchronous e-learning.

Overall, I feel good about learning in asynchronous e-learning

Online peer interaction (Yang & Chang (2012)

1. Learning through asynchronous e-learning increases the frequency of interaction with my classmates.

2. The use of asynchronous e-learning improves my understanding of classmates’ communication style.

3. With asynchronous e-learning, I am more willing to offer my opinion regarding how a course topic differs from other topics
